# Novel Circulating Tumour Cell-Related Risk Model Indicates Prognosis and Immune Infiltration in Lung Adenocarcinoma

**DOI:** 10.1155/2022/6521290

**Published:** 2022-05-29

**Authors:** Lu Liang, Yueying Liu, Shiyao Jiang, Jingjing Huang, Hua He, Li Shen, Li Cong, Yiqun Jiang

**Affiliations:** ^1^The Key Laboratory of Model Animal and Stem Cell Biology in Hunan Province, Hunan Normal University, Changsha, 410013 Hunan, China; ^2^School of Medicine, Hunan Normal University, Changsha, 410013 Hunan, China

## Abstract

**Background:**

Lung adenocarcinoma (LUAD) is the most common histological subtype of lung cancer (LC) and one of the leading causes of cancer-related death worldwide. LUAD has a low survival rate owing to tumour invasion and metastasis. Circulating tumour cells (CTCs) are precursors of distant metastasis, which are considered to adopt the characteristics of cancer stem cells (CSCs). Therefore, analysing the risk factors of LUAD from the perspective of CTCs may provide novel insights into the metastatic mechanisms and may help to develop diagnostic and therapeutic strategies.

**Methods:**

A total of 447 patients from TCGA dataset were included in the training cohort, and 460 patients from the GEO dataset were included in the validation cohort. A CTC-related-gene risk model was constructed using LASSO penalty–Cox analysis, and its predictive value was further verified. Functional enrichment analysis was performed on differentially expressed genes (DEGs), followed by immune correlation analysis based on the results. In addition, western blot, CCK-8 and colony formation assays were used to validate the biological function of *RAB26* in LUAD.

**Results:**

A novel in-silico CTC-related-gene risk model, named the CTCR model, was constructed, which successfully divided patients into the high- and low-risk groups. The prognosis of the high-risk group was worse than that of the low-risk group. ROC analysis revealed that the risk model outperformed traditional clinical markers in predicting the prognosis of patients with LUAD. Further study demonstrated that the identified DEGs were significantly enriched in immune-related pathways. The immune score of the low-risk group was higher than that of the high-risk group. In addition, *RAB26* was found to promote the proliferation of LUAD.

**Conclusion:**

A prognostic risk model based on CTC-related genes was successfully constructed, and the relationship between DEGs and tumour immunity was analysed. In addition, *RAB26* was found to promote the proliferation of LUAD cells.

## 1. Introduction

Lung cancer (LC) is one of the leading causes of cancer-related death worldwide. In addition, it is the malignant tumour with the highest morbidity and mortality in China [1]. Late diagnosis and distant metastasis are the two main reasons for its high mortality [2]. The morbidity of lung adenocarcinoma (LUAD) (the most common histological subtype of LC) is rapidly increasing [3, 4] owing to cigarette or tobacco abuse [5]. Although modern medicine has made significant progress in diagnosis and treatment, the 5-year survival rate of LC remains poor [6]. Early diagnosis and prompt treatment are particularly important to improve the long-term survival rate. In recent years, circulating tumour cells (CTCs) have gradually emerged as biomarkers for the early diagnosis of tumours.

CTCs are mainly derived from solid tumours, invade the blood circulation and are widely distributed in peripheral blood [7]. Most tumour cells die rapidly after entering peripheral blood circulation owing to hypoxia, immune recognition and other factors. Only a few CTCs with stem-cell-like characteristics survive [8], which can accumulate to form a tiny tumour thrombus, breaking through the vascular wall and invading distant organs. CTCs proliferate and metastasise to target organs with an appropriate microenvironment if they can evade recognition by the immune system. Jiang et al. found that CTCs can be detected in the peripheral blood of patients with early-stage cancer and those with benign diseases in addition to patients with distant metastasis of malignant tumours [9–11]. Their study suggests that CTC detection may be one of the reliable indicators for the early diagnosis and prediction of lymph node metastasis of LUAD. Jin et al. found that the overall survival (OS) and disease-free survival rates of patients with stage I or II non-small cell lung cancer (NSCLC) with >50 CTCs per 10 mL of peripheral blood were significantly reduced after tumour resection [12]. A recent study evaluating stage I tumours found that all patients with postoperative CTC elevation subsequently relapsed [13]. In addition, many studies have confirmed that CTCs are closely related to the prognosis of patients with malignant tumours such as prostate and breast cancers [14, 15]. Therefore, CTCs are of great significance for the early diagnosis, detection of recurrence and metastasis and prognosis evaluation of tumours.

In this study, we constructed an in silico risk model to analyse the correlation between CTC-related genes and the prognosis of patients with LUAD. The risk model, which was named the CTCR model, was based on CTC-related genes identified using the TCGA cohort and was validated in other cohorts. Furthermore, DEGs identified using the CTCR model were analysed via functional enrichment and immune correlation analyses. In addition, the biological function of *RAB26* in LUAD cells was verified via experiments. Overall, the novel CTCR model could guide the prognosis of patients with LUAD, and the immune correlation analysis provided new insights into the tumour microenvironment and immune infiltration of LUAD.

## 2. Materials and Methods

### 2.1. Patient Data Acquisition

**Figure 1:**
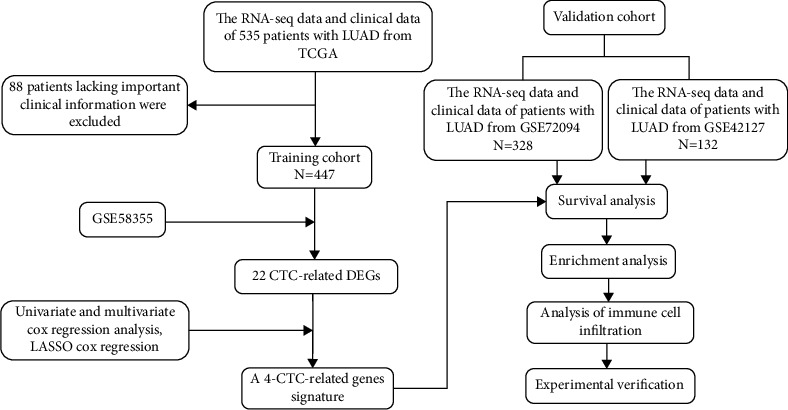
The flow chart of the study.

The RNA-sequencing (RNA-seq) data and clinical follow-up information of 535 patients with LUAD were downloaded from TCGA database (https://portal.gdc.cancer.gov/repository). Owing to the lack of critical clinical data for follow-up analysis (such as survival time, tumour stage, survival status and smoking history), 88 patient samples were excluded from the study. The data of 447 patients with LUAD were used to establish a training cohort to identify prognostic metastasis-related genes to construct a prognostic prediction model. Two LUAD gene expression datasets were downloaded from the GEO database (https://www.ncbi.nlm.nih.gov/GEO/). After screening patients according to the inclusion and exclusion criteria, 328 samples in the GSE72094 dataset and 132 samples in the GSE42127 dataset were, respectively, used to construct validation cohorts to verify the prognostic risk model. (Table 1, Figure 1).

### 2.2. Identification of CTC-Related DEGs in TCGA-LUAD Cohort

The GSE58355 dataset contains the gene expression profile of H1299 cells derived from a 4D tissue model, including the gene expression profiles of the primary tumour, CTCs and metastatic lesions. A Venn diagram was generated to analyse the GSE58355 dataset, and 26 DEGs related to CTCs, primary tumour and metastasis lesions were identified in LUAD (|log FC|>1.5, *p* < 0.05). Furthermore, the expression of the 26 DEGs in 347 normal tissues from the GTEx database and 447 cancer tissues from TCGA-LUAD cohort was analysed using the R package ‘limma'. A heat map was drawn using the ‘gplots' R package to visualise the expression of DEGs in the clinical subgroup of 447 TCGA-LUAD samples. A Sankey diagram was drawn to analyse the association of DEGs with clinical features and prognosis (|r|>0.15, *p*<0.05). In addition, a heat map was generated to analyse the correlation among CTC-related DEGs.

### 2.3. Construction and Validation of the CTCR Model

Univariate and multivariate regression analyses were performed on DEGs to evaluate their role in predicting the OS of patients. Subsequently, candidate genes were identified using LASSO analysis with 10 cross-validations to construct the prognostic risk model. The risk score of each sample was calculated using the following formula: risk score = sum (each candidate gene expression × corresponding LASSO regression coefficient) [16].

Kaplan–Meier (KM) survival curves were plotted using the R package ‘survival', risk plots were generated using the R package ‘ggrisk' and principal component analysis (PCA) was performed using the R package ‘stats'. The R package ‘survival ROC' was used for time-dependent receiver operating characteristic (ROC) curve analysis and to calculate the area under the curve (AUC) values at 1, 3 and 5 years.

The two validation cohorts were divided into the high- and low-risk groups based on their median risk score. KM survival curves, ROC curves and the expression of candidate genes were analysed in both cohorts after confirming the reliability of the risk model through PCA.

### 2.4. Enrichment Analysis

The ‘limma' R package was used to identify DEGs between the high- and low-risk groups in TCGA-LUAD cohort (|log2FC| >1*, p* <0.05). Gene Ontology (GO) was used to analyse the biological processes, molecular functions and cellular components. Kyoto Encyclopedia of Genes and Genomes (KEGG) was used to identify the related signalling pathways. Furthermore, DEGs were subjected to gene set enrichment analysis (GSEA) using the ‘gseaplot2' package to analyse their biological functions and signal transduction pathways.

### 2.5. Assessment of the Immune Microenvironment

The ESTIMATE, immune and stromal scores were evaluated using the R package ‘estimate' [17, 18]. Violin plots were generated using the R package ‘ggplot2' to evaluate the scores of 6 immune checkpoints.

### 2.6. Analysis of the Association between *RAB26* and Immune Cells

Samples from the training cohort were analysed using the ssGSEA algorithm, and the enrichment scores of 24 immune cells were demonstrated in a box plot. A lollipop chart generated using the ‘ggplot2' R package demonstrated the correlation between *RAB26* and immune cells. Subsequently, 8 cells with the most significant correlation coefficient were selected for generating a scatter plot (|r| >0.15, *p <*0.01).

### 2.7. Cell Culture, Plasmids and shRNAs

The LUAD cell lines H1299 (ATCC: CRL-5803™) and A549 (ATCC: CCL-185™) were obtained from American type culture collection (ATCC). H1299 cells were cultured in RPMI 1640 (Gibco, Carlsbad, CA, United States), whereas A549 cells were cultured in DMEM/F12 (Hyclone, Logan, UT, United States). Both media were supplemented with 10% (v/v) fetal bovine serum (FBS, Biological Industries, Israel) and antibiotics, and all cells were maintained at 37°C in an atmosphere of 5% CO_2_. Both cell lines yielded a negative result for mycoplasma contamination. They were passaged <10 times after their initial revival from frozen stocks and were authenticated by performing short tandem repeat profiling before use.

A lentiviral vector overexpressing *RAB26* was generated by cloning *RAB26* cDNA into a pLVX-EF1*α*-IRES-Puro vector (catalogue no. 631988; Clontech, Mountain View, CA, USA) using the restriction enzymes EcoRI and BamHI (Takara).

Lentiviral shRNA vectors targeting human *RAB26* and a scramble control vector were purchased from Genechem (https://www.genechem.com.cn; Shanghai, China). All plasmid vectors were verified via sequencing. The target sequences used are as follows:

Mock: 5′-CCTAAGGTTAAGTCGCCCTCG-3′

sh*RAB26*#1: 5′-CCGGCTGCATGATTACGTTAA-3′

sh*RAB26*#2: 5′-ACAGAAGGCTTCACTGCTAAT-3′

All transfection procedures were performed according to the manufacturer's instructions.

### 2.8. Western Blot

Cells were digested, collected and washed twice with cold phosphate-buffered saline (PBS). Thereafter, they were lysed on ice using lysate buffer (#87787, Thermo Fisher) with a protease inhibitor (#P1010, Beyotime) for 30 min and centrifuged at 15,000 g at 4°C for 15 min. The supernatants were collected as whole cell lysates, and protein concentration was determined via BCA assay (#E112-01, Vazyme). A quantity of 50 *μ*g of total protein and the following antibodies were used for western blot: mouse monoclonal anti-human *β*-actin antibody (#AF7018, Affinity), rabbit anti-human *RAB26* antibody (#14284-1-AP, Proteintech), goat anti-rabbit antibody (#S0001, Affinity) and goat anti-mouse antibody (#A21010, Abbkine).

### 2.9. Cell Proliferation Assay

Stable *RAB26*-overexpressing A549 cells and *RAB26*-knockdown H1299 cells were seeded in RPMI-1640 or DMEM/F12 medium (100 *μ*L) in 96-well plates at a density of 2 × 10^3^ cells/well. Cell viability was measured at 0, 24, 48 and 72 h using Cell Counting Kit-8 (CCK-8, Code: A311-01, Vazyme, Nanjing, China) according to the manufacturer's instructions. Briefly, the Cell Counting Kit-8 solution was added to cells (10 *μ*L/well) and incubated for 2 h. The absorbance was at 450 nm measured using a microplate reader (Synengy2, Bio-Tek, USA), and the GraphPad Prism (version 9.1.0) software was used for plotting the proliferation curve.

### 2.10. Colony Formation Assay

Approximately 200 cells/well were inoculated in a 6-well plate and cultured in a medium. After 2 weeks, the cells were fixed with methanol for 15 min and stained with 0.1% crystal violet for 20 min. The number of visible colonies was calculated using the ImageJ software, and the colonies with a diameter of >0.05 mm were scored.

### 2.11. Statistical Analysis

The chi-square test was used to analyse differences in the proportion of clinical features. Univariate and multivariate Cox regression analyses were performed to determine the independent prognostic factors of OS. Time-dependent ROC curve analysis was performed to examine the prediction accuracy of the prognostic model. KM analysis was performed to assess OS. Wilcoxon test was used to compare the proportion of tumour-infiltrating immune cells and the expression of immune checkpoint molecules between the high- and low-risk groups. Spearman correlation coefficients were used for correlation analyses. All experiments were performed in triplicate. All statistical analyses were performed using the SPSS Statistics (version 23.0) or R (version 4.0.5) software. In addition, *p*-values generated via bioinformatic analyses were adjusted via multiple testing correction using the Benjamini–Hochberg procedure, with *p-*values of <0.05 being considered statistically significant. The statistical methods and algorithms used are described in the corresponding sections.

## 3. Results

### 3.1. A Total of 22 DEGs Associated with CTCs and Metastasis Were Identified in TCGA Cohort

**Figure 2:**
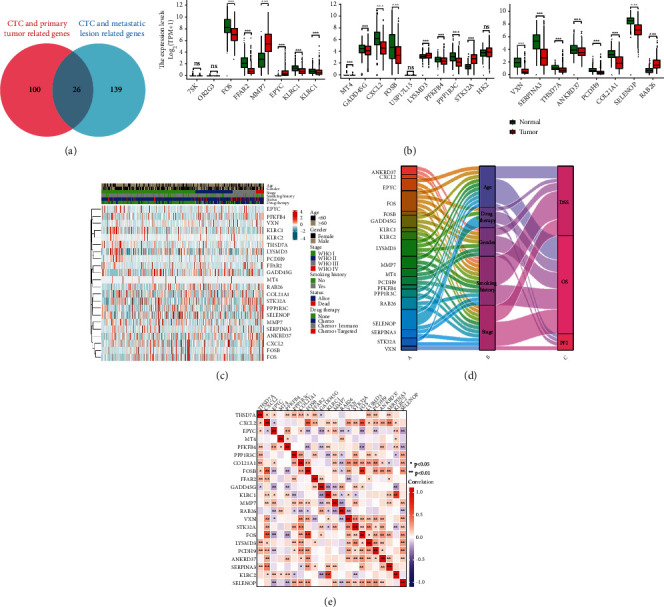
Identification and analysis of CTC-related DEGs in patients with LUAD. (a) Venn diagram demonstrating genes related to CTCs, primary tumour and metastasis. (b) The expression level of 26 overlapping genes in tumour samples in TCGA-LUAD cohort (n = 447) and normal samples in the GTEx dataset (n = 347). (c) Unsupervised clustering of DEG expression in TCGA cohort. The age, sex, tumour stage, smoking history, survival status and drug therapy are used as patient annotations. (d) Sankey diagram demonstrating the degree of association among DEGs, clinical features and prognosis. (e) Heat map demonstrating the correlation among 22 genes in TCGA-LUAD cohort. The asterisks represent the statistical *p*-value (∗*p* < 0.05; ∗∗*p* < 0.01; ∗∗∗*p* < 0.001).

On analysing the RNA-seq data of patients with LUAD in the GSE58355 dataset, 126 genes related to CTCs and primary tumours and 165 genes associated with CTCs and metastasis were identified. The interaction among these genes was analysed, revealing 26 DEGs (Figure 2(a),|logFC|> 1.5, *p* < 0.05). Of these 26 DEGs, 22 were significantly differentially expressed in normal and tumour tissues (Figure 2(b)). A heat map was generated to demonstrate the expression of the 22 DEGs among patients with different clinical characteristics such as age, sex, tumour clinical stage, smoking history, survival status and treatment methods (Figure 2(c)). A Sankey diagram (Figure 2(d)) was generated to understand the relationship between these genes and the clinical characteristic of patients, which revealed that most DEGs were associated with age, smoking history and tumour staging. In particular, smoking history and tumour stage were significantly correlated with prognosis. In addition, some correlation was observed among the DEGs. For example, *RAB26* had a significantly negative correlation with *EPYC* and *MMP7* (Figure 2(e), (*p)<* 0.01). Additionally, the cluster dendrogram reflected the similarity among 22 DEGs (Supplementary Figure S1). These results revealed that the 22 DEGs were closely related to CTCs and metastasis in TCGA-LUAD and GSE58355 datasets.

### 3.2. The CTCR Model Was Constructed Using 4 Genes

**Figure 3:**
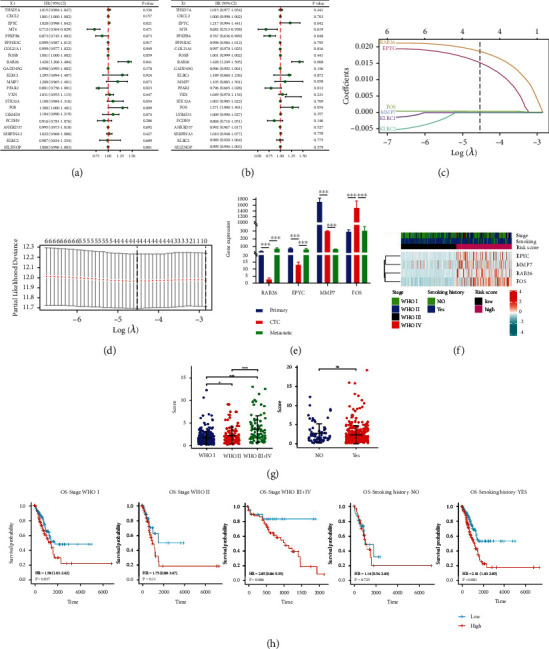
Establishment of the CTCR model in TCGA-LUAD cohort (n = 447). (A, B) Univariate and multivariate Cox regression analyses were used to examine the correlation between DEGs and OS. (C) LASSO coefficient profiles of 6 DEGs. (D) Cross-validation revealed an optimum parameter of 4 in the LASSO model. (E) The relative expression of 4 risk genes in the primary tumour, CTCs and metastasis. (F) Unsupervised clustering of the 4 candidate genes using the tumour stage, smoking history and risk score as patient annotations. (G) Dot chart reflecting the risk scores of samples grouped based on the clinical stage and smoking history (H) Prognostic value in the high- and low-risk groups of different clinical subgroups (tumour stages and smoking history). The asterisks represent the statistical *p*-value (∗*p* < 0.05; ∗∗*p* < 0.01; ∗∗∗*p* < 0.001).

Univariate and multivariate Cox regression analyses were used to evaluate the hazard ratio (HR) and *p*-value of 22 DEGs **(**Figures 3(a) and 3(b)**)**. Based on the abovementioned datasets, 6 genes (*EPYC*, *RAB26*, *MMP7*, *FOS*, *KLRC1* and *KLRC2*) were selected for subsequent analysis, with the threshold set as |logFC|values of >2.0 and *p-*values of <0.001. LASSO penalty–Cox analysis indicated that the deviation was smallest when the number of genes in the model was 4 **(**Figures 3(c) and 3(d)**)**. Therefore, the optimal candidate genes *RAB26*, *EPYC*, *MMP7* and *FOS* were used to build a prognostic signature, and the risk score was calculated using the following formula: risk score = 1.879 × *RAB26*+ 1.540 × *EPYC*+ 0.018 × *MMP7*+ 0.030 × *FOS*.

Patients in the training cohort were divided into the high- and low-risk groups based on the median risk score. Furthermore, the relative expression of the 4 risk genes dramatically changed in the primary tumour, CTCs and metastasis, with no fixed trend (Figure 3(e)). The heat map demonstrated that the expression of the 4 risk genes was significantly higher in the high-risk group (Figure 3(f)), and the data are shown in Supplementary Data 1. Subsequently, the training cohort was divided into different subgroups based on the smoking history (Yes, No) and disease stage (WHO I, WHO II and WHO III–IV) of patients. The dot plot showed that the risk score was higher in patients with advanced-WHO-stage cancer, and no significant difference was observed in the risk score between patients who smoked and those who did not smoke (Figure 3(g)). The KM curve indicated that OS was prominently lower in the high-risk group than in the low-risk group in different subgroups (Figure 3(h)). Besides, The KM curves with high and low expression groups for the four DEGs were presented in Supplemental Figures S2. Therefore, a novel in silico CTC-related risk model, named the CTCR model, was successfully constructed based on 4 genes, including *RAB26*, *EPYC*, *MMP7* and *FOS*.

### 3.3. High Predictive Ability of the CTCR Model Was Verified in the Training Cohort

**Figure 4:**
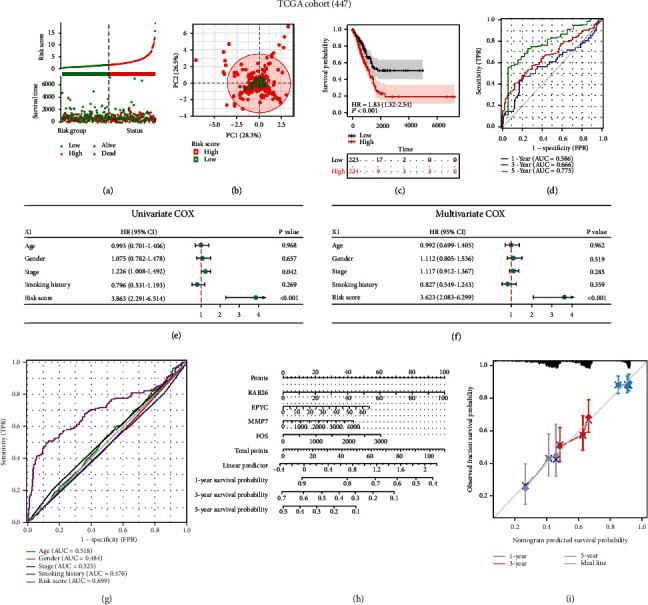
Evaluation of the CTCR model in TCGA-LUAD cohort (n = 447). (a) Survival status and risk scores in the training cohort. (b) PCA analysis of grouped samples in the training cohort. (c) KM curves demonstrating longer OS in the low-risk group. (d) AUC values of time-dependent ROC curves verify the predictive accuracy of the risk score. (E, F) Univariate and multivariate Cox analyses were performed to examine the correlation among clinical features, risk scores and OS. (G) Comparison of ROC curves among patients with different clinical characteristics, such as age, sex and stage, and risk scores in the training cohort. (H) Nomogram based on the 4 CTC-related genes quantitatively predicts the survival of patients with LUAD. (i) Calibration plot for internal validation of the nomogram.

The risk curve and prognosis distribution of patients in the training cohort revealed that patients in the low-risk group had a higher survival probability than those in the high-risk group (Figure 4(a)). PCA showed that the prognosis of the low- and high-risk groups was different, which further indicated the effectiveness of the CTCR model (Figure 4(b)). KM analysis showed that the OS of patients was significantly lower in the high-risk group than in the low-risk group (Figure 4(c), (*p)<* 0.001). The AUC value was 0.586 at 1 year, 0.666 at 3 years and 0.775 at 5 years (Figure 4(d)), which suggested that the risk model had an excellent ability to predict the long-term prognosis of patients with LUAD.

Univariate Cox regression analysis suggested that the pathological stage (HR = 1.226, 95% CI = 1.008–1.492, *p <0.05*) and risk scores (HR = 3.863, 95% CI = 2.291–6.514, *p <* 0.001) were prognostic factors for patients with LUAD (Figure 4(e)), whereas multivariate Cox regression analysis showed that only the risk score could be considered an independent prognostic indicator for patients with LUAD (Figure 4(f), HR = 3.623, 95% CI = 2.083–6.299, *p <* 0.001). Furthermore, the AUC values of the risk score (Figure 4(g)) and the combination of clinical characteristics and risk score (Supplementary Figure S3) were 0.699 and 0.713, which were higher than those of other clinical characteristics.

A prognostic nomogram was constructed to predict 1-, 3- and 5-year survival in TCGA cohort (Figure 4(h)). As shown in Figure 4(h), the expression of each prognostic gene in the risk model had a corresponding score The total score was obtained by adding the scores of all variables, and the survival probability of patients with LUAD was calculated according to the total score. Furthermore, the calibration plots of the nomogram suggested that the bias-corrected line was close to the ideal line, which indicated definitive agreement between the actual and predicted 1-, 3- and 5-year survival probabilities (Figure 4(i)). Therefore, the CTCR model had an excellent ability to predict the long-term survival of patients with LUAD.

### 3.4. Predictive Ability of the CTCR Model Was Verified in Validation Cohorts

**Figure 5:**
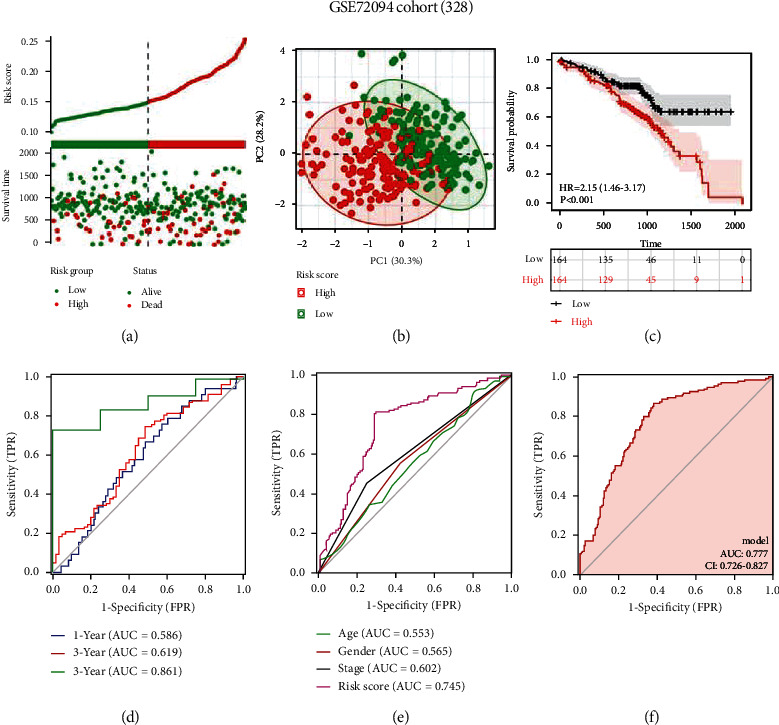
Validation of the CTCR model in the GSE72094 dataset (n = 328). (a) Survival status and risk scores in the validation cohort. (b) PCA analysis of grouped samples in the validation cohort. (c) KM curves demonstrating longer OS in the low-risk group. (d) AUC values of time-dependent ROC curves verify the predictive accuracy of the risk score. (e) Comparison of ROC curves among patients with different clinical characteristics, such as age, sex and stage, and risk scores in the validation cohort. (f) ROC curve based on risk scores and other prognostic factors.

To test the robustness of the CTCR model, GSE72094 and GSE42127 datasets were used as validation cohorts. In both cohorts, the expression of *RAB26* and *EPYC* was higher in the high-risk group as shown in the heat map (Supplementary Figure S4). The risk scores and survival status of samples in both cohorts are shown in Figure 5(a) and Supplementary Figure S5A. In addition, their grouping was validated (Figure 5(b), Supplementary Figure S5B). Subsequently, the KM survival curve showed a poor prognosis for high-risk groups (Figure 5(c), Supplementary Figure S5C). In the GSE72094 and GSE42127 cohorts, the CTCR model was found to have good ability to predict the survival of patients with LUAD, particularly 5-year OS (Figure 5(d), Supplementary Figure S5D). Furthermore, the risk model had higher predictive accuracy than traditional clinical characteristics (Figure 5(e), Supplementary Figure S5E). The AUC value of the combination of clinical characteristics and risk score was 0.777 in the GSE72094 cohort and 0.830 in the GSE42127 cohort, which was higher than that of other clinical characteristics (Figure 5(f), Supplementary Figure S5F). Overall, these results were consistent with those observed in the training cohort and indicated the reliability of the CTCR model.

### 3.5. Enrichment Analysis Showed That DEGs Were Significantly Correlated with Tumour Immunity

**Figure 6:**
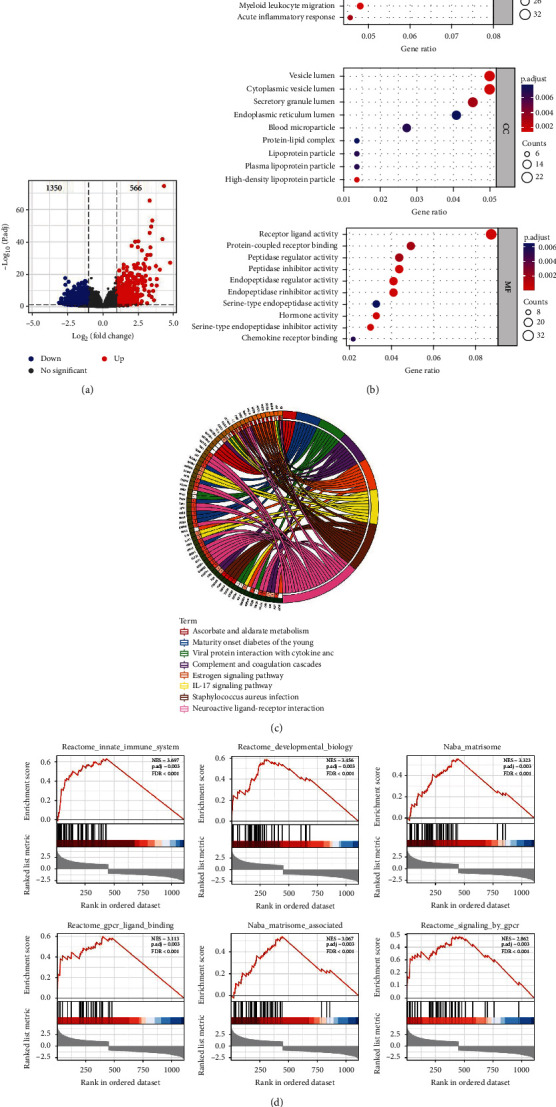
Enrichment analysis of DEGs in TCGA-LUAD cohort (n = 447). (a) Volcano plot depicting DEGs in the high- and low-risk groups in the training cohort (including 566 upregulated and 1350 downregulated genes). (b) GO analysis was used to determine critical biological processes, cellular components and molecular functions, indicating that the DEGs were significantly enriched in immune-related processes. (c) Chord diagram demonstrating the results of KEGG analysis, indicating that the DEGs were considerably enriched in immune-related signalling pathways. (d) GSEA of the high- and low-risk groups based on the CTCR model.

Based on the CTCR model, DEGs (low-risk versus high-risk group) were identified using data from TCGA-LUAD cohort, with a total of 566 upregulated and 1350 downregulated genes (Supplementary Data 2; Figure 6(a), |log2FC| >1, *p <*0.05). These DEGs were used for subsequent analysis, and the relevant data are presented in Supplementary Data 3 and 4. GO pathway enrichment analysis showed that the DEGs were significantly enriched in many immune-related biological processes, such as humoral immune response, antibacterial humoral response, defence against bacterial infection, cell chemotaxis and acute inflammatory response (Figure 6(b)). KEGG pathway analysis also revealed that the DEGs were enriched in immune-related pathways, such as the IL-17 signalling pathway (Figure 6(c), Supplementary Figure S6). GSEA revealed that the DEGs were mainly enriched in 6 pathways, including ‘innate immune system' (Figure 6(d)), which contributes to tumour control by activating DCs [19]. Enrichment analysis showed that the DEGs were significantly correlated with immune-related functions and signalling, which indicated the role of DEGs in tumour immunity. However, the association between the CTCR model and tumour immunity warrants further investigation.

### 3.6. Immune Cell Infiltration Was Analysed Based on the CTCR Model

**Figure 7:**
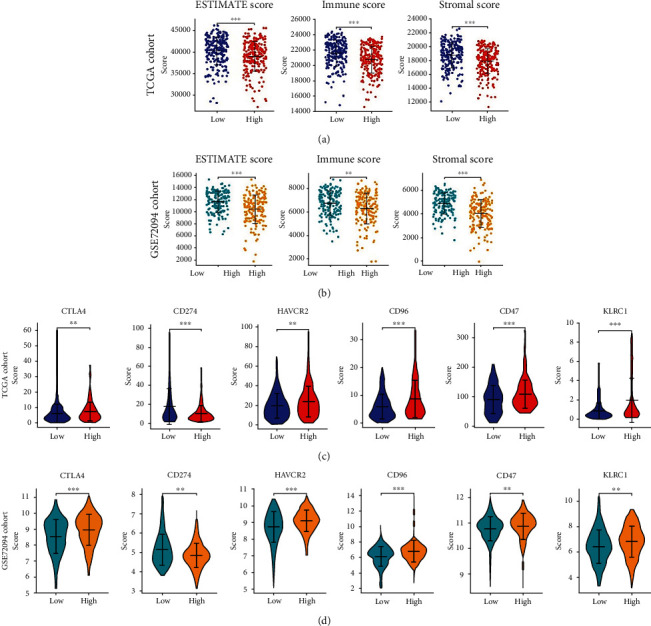
Immune cell infiltration landscape of LUAD. (A, B) ESTIMATE, immune and stromal scores of high- and low-risk patients in TCGA (a) (n = 447) and GSE72094 (b) (n = 328) datasets. (C, D) The expression levels of immune checkpoint molecules in the high- and low-risk groups in TCGA (c) and GSE72094 (d) datasets. The asterisks represent the statistical *p*-value (∗*p* < 0.05; ∗∗*p* < 0.01; ∗∗∗*p* < 0.001).

Given the significant enrichment of DEGs in immune function and related pathways, we further analysed the correlation between immunity and the CTCR model. In the assessment of the tumour microenvironment, the ESTIMATE algorithm demonstrated that the high-risk group in TCGA cohort was significantly associated with low ESTIMATE, immune and stromal scores. The same results were obtained in the two validation cohorts (Figures 7(a) and 7(b), Supplementary Figure S7A). Subsequently, 6 common immune checkpoints were selected, and the expression of CTLA4, CD96, CD47 and KLRC1 was found to be higher in the training and validation cohorts. This finding indicated that patients with low risk scores might benefit from immune checkpoint inhibitor (ICI) therapy [20] (Figures 7(c) and 7(d), Supplementary Figure S7B).

### 3.7. Association Analysis between *RAB26* and Immune Cells

**Figure 8:**
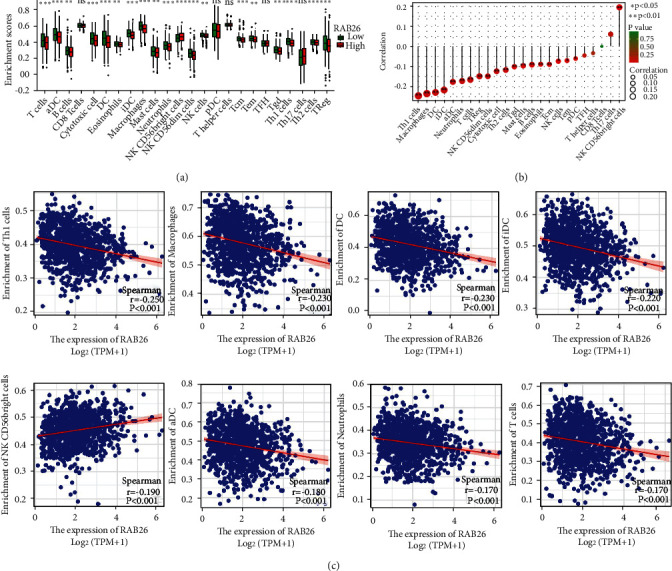
Correlation between *RAB26* expression and immune cells in TCGA-LUAD cohort (n = 447). (a) Box plot demonstrating the infiltration levels of immune cells in the high-*RAB26*-expression versus the low-*RAB26*-expression group. (b) Lollipop chart demonstrating the correlation between *RAB26* and 24 immune cell types. (c) Correlation scatter plot of 8 cells with the largest correlation coefficient with *RAB26*. The asterisks represent the statistical *p*-value (∗*p* < 0.05; ∗∗*p* < 0.01; ∗∗∗*p* < 0.001).

*RAB26* was selected for further immune infiltration analysis owing to its highest LASSO regression coefficient. Based on the median expression level of *RAB26*, samples in the training cohort were divided into two groups. ssGSEA was performed to assess the immune status of patients in TCGA-LUAD cohort, and it was found that the patients with high expression of *RAB26* had more abundant immune-infiltrating cells (Figure 8(a)). In addition, *RAB26* expression was negatively correlated with CD56^bright^ NK cells and positively correlated with other cells (Th1 cells, Macrophages, DC, iDC, aDC, Neutrophils, T cells, TReg, NIL CD56dim cells, Cytotoxic cells, Th2 cells, Tgd, Mast cells, B cells, Eosinophils, Tcm, NIK cells, Tem, pDC); however, the correlation between *RAB26* expression and TFH, T helper, CD8 T and Th17 cells was not significant (Figure 8(b)). Subsequently, eight immune cells with the highest correlation coefficients were selected (Th1, macrophages, DCs, iDCs, CD56^bright^ NK cells, aDCs, neutrophils and T cells) for generating a scatter plot demonstrating the correlation between *RAB26* expression and immune infiltration (Figure 8(c)). These results suggested that the group with high *RAB26* expression had higher levels of immune cell infiltration, which also implied that high *RAB26* expression might be associated with a poor prognosis in patients with LUAD.

### 3.8. *RAB26* Promoted the Proliferation of LUAD Cells

**Figure 9:**
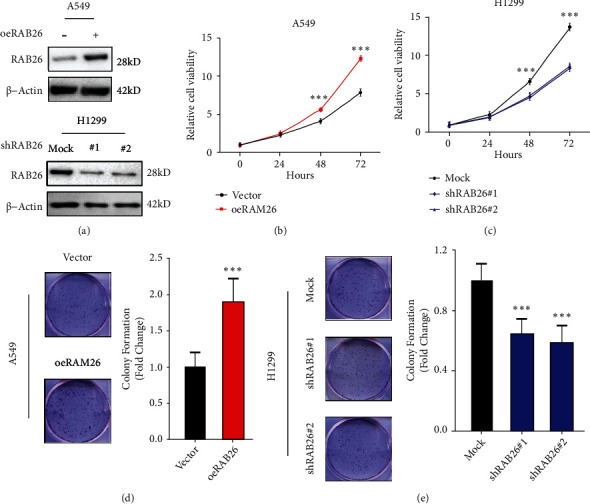
*RAB26* promoted LUAD cell proliferation. (a) Western blot was performed to confirm the overexpression of *RAB26* in A549 cells (top) and its knockdown in H1299 cells (bottom). (b, c) Cell proliferation assay of *RAB26*-overexpressing A549 cells, *RAB26*-knockdown H1299 cells and corresponding normal control cells. (d, e) Colony formation assay validated the promoting effect of *RAB26* on the proliferation of H1299 and A549 cells. The asterisks represent the statistical *p*-value (∗*p* < 0.05; ∗∗*p* < 0.01; ∗∗∗*p* < 0.001).

A study [21] involving the established human NSCLC cell lines showed that the expression of *RAB26* was higher in H1299 cells and lower in A549 cells. In this study, *RAB26* was overexpressed in A549 cells and knocked down in H1299 cells using a lentivirus to examine whether *RAB26* plays an essential role in the progress of LUAD. Western blot showed that *RAB26* was successfully overexpressed in A549 cells and knocked down in H1299 cells (Figure 9(a)). CCK-8 assay revealed that overexpression of *RAB26* promoted the proliferation of A549 cells and knockdown of *RAB26* inhibited the proliferation of H1299 cells (Figures 9(b) and 9(c)). In addition, the colony formation assay revealed that *RAB26* promoted LUAD cell proliferation (Figures 9(d) and 9(e)).

## 4. Discussion

CTCs capable of surviving in the blood circulation have CSC-like features and are a sign of cancer recurrence and distant metastasis. Considering the advantages of CTCs in early tumour diagnosis, CTC-related genes were screened in this study based on the data of patients with LUAD. The identified DEGs were used to construct an in silico risk signature, named the CTCR model. Furthermore, KM survival and ROC analyses were performed in the training and validation cohorts. The 5-year AUC values of both validation cohorts exceeded 0.8, indicating that the CTCR model had a superior ability to predict the long-term survival of patients with LUAD. Although two validation sets were used to verify the credibility of the model, further investigation is required to validate the results owing to the use of public databases.

Four genes (*RAB26*, *EPYC*, *MMP7* and *FOS*) were selected to construct the risk signature. These genes are associated with tumour development; some have been reported in studies on LC. For example, in a study, overexpression of *RAB26* in pulmonary microvascular endothelial cells inhibited LPS-induced apoptosis through the TLR4 pathway [22]. This finding suggests that *RAB26* promotes the occurrence and development of LC by regulating apoptosis [21]. In addition, *RAB26* benefits the proliferation of nasopharyngeal carcinoma cells [23], which is consistent with the results of the cell proliferation assay performed in this study. Furthermore, upregulation of *MMP7* promotes the migration and invasion of LUAD [24]. Studies on human hepatoma cell lines have shown that c-*FOS* plays an essential role in cell migration [25]. Related studies have also indicated that *EPYC* promotes intestinal metastasis of ovarian cancer and is significantly associated with a poor prognosis [26].

Subsequent analyses showed that DEGs between the high- and low-risk groups were significantly enriched in immune-related pathways and functions. Considering that the tumour microenvironment and immune cell infiltration are correlated with the prognosis of cancer [27], it is necessary to explore the tumour immune microenvironment of LUAD. Each immune cell type plays a different role. For example, Jonge et al. reported that the abundance of CD56^bright^ NK cells was negatively correlated with OS and distant metastases in human melanoma [28]. In this study, the infiltration levels of CD56^bright^ NK cells were found to be higher in the high-*RAB26*-expression group (Figure 8(a)). On analysing the correlation between *RAB26* and 24 types of immune cells, only CD56^bright^ NK cells were found to be positively correlated with *RAB26* expression. This finding suggested that high *RAB26* expression indicated tumour metastasis and a poor prognosis. In addition, *RAB26* expression was negatively correlated with cells that mediate immune surveillance, such as NK cells (Figure 8(b)). NK cells play a key role in destroying CTCs and avoiding cancer metastasis [29]. When immune escape occurs, the number and cytotoxic activity of NK cells are reduced [30]. Therefore, we concluded that *RAB26* might help CTCs to evade immune surveillance and promote LUAD progression by regulating immune cell infiltration in the tumour microenvironment.

An immunosuppressive tumour microenvironment is a sanctuary for primary tumours [31]. However, as the tumour cells proliferate, some cells slough off the edges of a tumor and enter the circulatory system, only a few CTCs survive in the active immune surveillance environment [8, 32]. These surviving CTCs can accumulate to form circulating tumour microemboli (CTM). The crosstalk between CTCs and other components in CTM creates a tumour microenvironment favourable for cancer cell survival. During this process, genetic alterations or abnormal expression of some genes and physiological changes affect the survival of CTCs in peripheral blood, thus promoting new distant metastases [33]. Therefore, CTC-related genes (genes related to CTCs, primary tumours and metastatic lesions) may mediate immune evasion of CTCs and promote the metastasis, proliferation or drug resistance of cancer cells [34]. Epithelial–mesenchymal transition (EMT) is a process by which cells lose their epithelial properties and gain mesenchymal characteristics [35]. It leads to the production of CTCs from the primary tumour and promotes the metastatic ability of CTCs in the circulation [36]. In addition, EMT promotes the invasive growth and distant metastasis of LC cells [37]. Numerous studies have proven that signal transducer and activator of transcription 3 (STAT3) affect EMT in cancer [38–40]. Coincidentally, RAB26 was identified as a novel target gene for SNRPB-mediated RNA maturation and demonstrated that RAB26 partly contributes to the oncogenic functions of SNRPB in NSCLC [21]. Therefore, we speculate that RAB26, one of the CTC-related DEGs identified in this study, promotes the development and metastasis of LUAD and helps CTCs to evade immune-mediated killing by inducing EMT. These CTCs survive in the inhospitable circulatory microenvironment and colonise distant sites [41].

In conclusion, a prognostic risk model based on 4 CTC-related genes was established, and its predictive value was evaluated. The results provide new insights into the prognostic prediction of LUAD, which may help to develop diagnostic and individualised treatment strategies. Furthermore, in this study, DEGs related to CTCs and metastasis were screened in patients with LUAD. Because metastasis significantly affects the prognosis of patients, DEGs identified in this study should be further analysed and verified. *RAB26*, as a gene related to CTCs, primary tumours and metastatic lesions, promoted the proliferation of LUAD cells. Therefore, *RAB26* may be a candidate target for the treatment of patients with LUAD [42]. However, the specific mechanisms and pathways of *RAB26* affecting the tumour microenvironment warrant further investigation.

## 5. Conclusion

A prognostic risk model was constructed based on CTC-related genes, which could predict the prognosis of patients with LUAD. It was especially reliable in predicting long-term prognosis. In addition, the identified DEGs were closely associated with tumour immunity, and *RAB26* was found to promote the proliferation of LUAD cells. This study may provide new insights into the diagnosis and treatment of LUAD.

## Figures and Tables

**Table 1:** Clinical characteristics of patients in the training cohort and validation cohort.

Variables	Training cohort		Validation cohorts			
	TCGA(n = 447)		GSE72094(n = 328)		GSE42127(n = 132)	
	No.	%	No.	%	No.	%
Age	—	—	—	—	—	—
Median (years)	70.0	—	69.7	—	65.8	—
< 60	128	28.6	46	14.0	35	26.5
≥ 60	319	71.4	282	86.0	97	73.5
Gender	—	—	—	—	—	—
Male	207	46.3	156	47.6	67	50.8
Female	240	53.7	172	52.4	65	49.2
Stage	—	—	—	—	—	—
WHO I	259	57.9	218	66.5	89	67.4
WHO II	103	23.1	53	16.2	22	16.7
WHO III	64	14.3	46	14.0	20	15.1
WHO IV	21	4.7	11	3.3	1	0.8

## Data Availability

The following information was supplied regarding data availability: The data is available at the TCGA database (https://portal.gdc.cancer.gov/) and NCBI GEO: GSE72094 and GSE42127.

## Supplementary Materials

Supplementary Materials The following are the supplementary material to this article: Supplementary Data 1: Data for Figure 3(f) heat map. Supplementary Data 2: Data for Figure 6(a) volcano plot. Supplementary Data 3: Data for Figure 6(b), S6 GO and KEGG. Supplementary Data 4: Data for Figure 6(c) circle diagram. Supplementary Figures: Supplementary Figure S1: The cluster tree shows the similarity between 22 DEGs. Supplementary Figure S2: KM curve displays the survival possibility for the 4 candidate genes' low and high expression groups. Supplementary Figure S3: ROC curve considered risk score and other prognostic factors in TCGA-LUAD data. Supplementary Figure S4: Unsupervised clustering of 4 DEGs expressions in GSE72094 (A) and GSE42127 (B), while the tumor stage, smoking history, and risk score are used as patient annotations. Supplementary Figure S5: Validation of the CTCR Model in the GSE42127 dataset (n = 132). Supplementary Figure S6: Bar chart derived from KEGG analysis. Supplementary Figure S7: The immune cell infiltration landscape in GSE42127 dataset (n = 132).
