# Supplementary material for: Novel Circulating Tumour Cell-Related Risk Model Indicates Prognosis and Immune Infiltration in Lung Adenocarcinoma
Source: J Immunol Res. 2022 May 29;2022:6521290. doi: 10.1155/2022/6521290 (PMC9168189; doi:10.1155/2022/6521290)
Supplement: Supplementary Materials — The following are the supplementary material to this article: Supplementary Data 1: Data for Figure 3(f) heat map. Supplementary Data 2: Data for Figure 6(a) volcano plot. Supplementary Data 3: Data for Figure 6(b), S6 GO and KEGG. Supplementary Data 4: Data for Figure 6(c) circle diagram. Supplementary Figures: Supplementary Figure S1: The cluster tree shows the similarity between 22 DEGs. Supplementary Figure S2: KM curve displays the survival possibility for the 4 candidate genes' low and high expression groups. Supplementary Figure S3: ROC curve considered risk score and other prognostic factors in TCGA-LUAD data. Supplementary Figure S4: Unsupervised clustering of 4 DEGs expressions in GSE72094 (A) and GSE42127 (B), while the tumor stage, smoking history, and risk score are used as patient annotations. Supplementary Figure S5: Validation of the CTCR Model in the GSE42127 dataset (n = 132). Supplementary Figure S6: Bar chart derived from KEGG analysis. Supplementary Figure S7: The immune cell infiltration landscape in GSE42127 dataset (n = 132). [file 6521290.f1.zip › Supplementary Figures.docx]

**
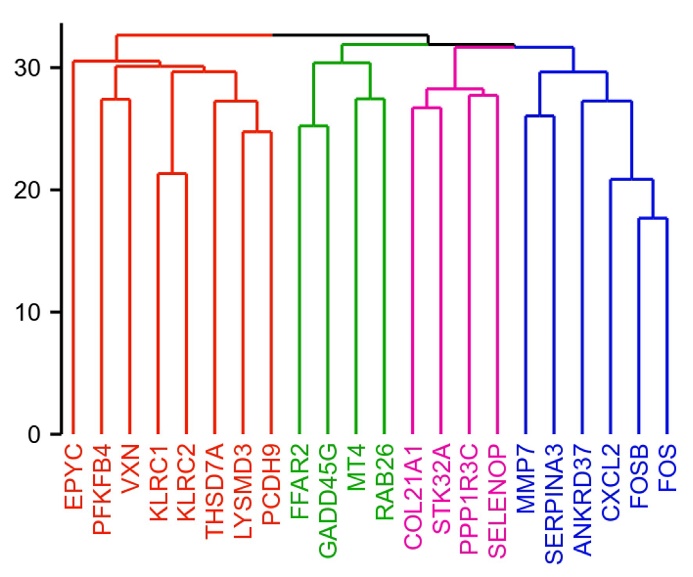
**

**Supplementary Figure S1** The cluster tree showing the similarity between 22 DEGs.


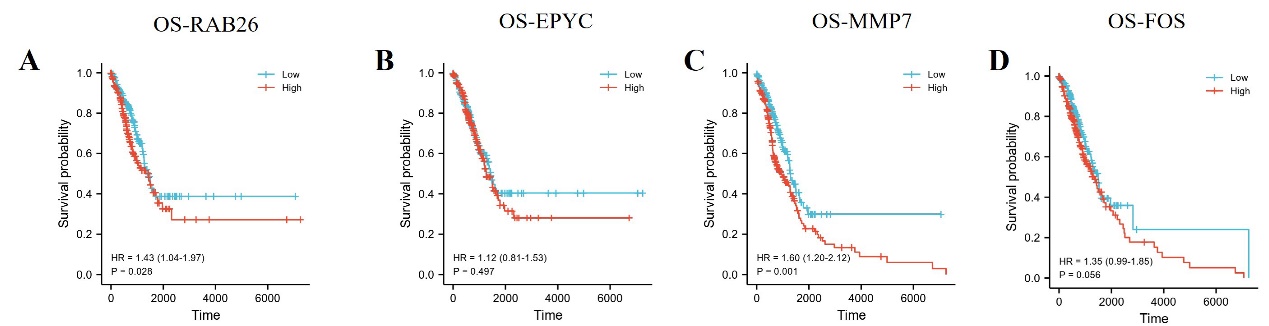


**Supplementary Figure S2** KM curve displaying the survival possibility for the 4 candidate genes' low and high expression groups.


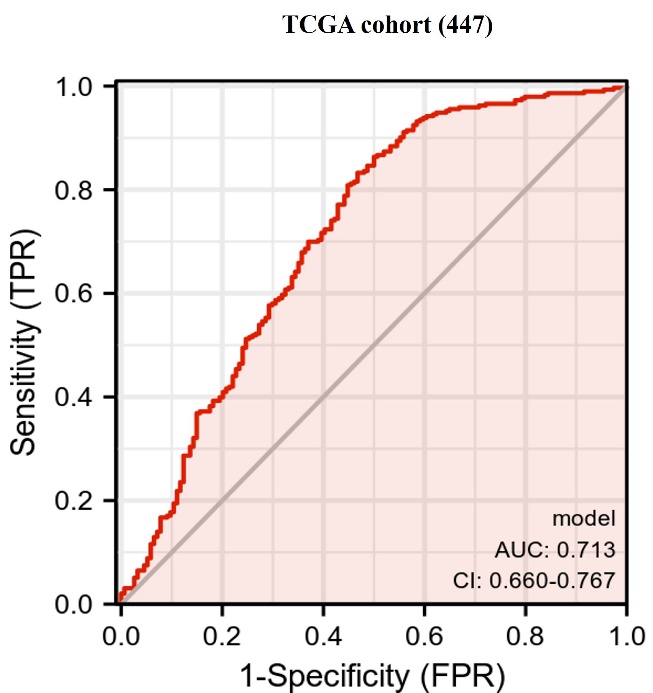


**Supplementary Figure S3** ROC curve based on risk scores and other prognostic factors in TCGA-LUAD data.


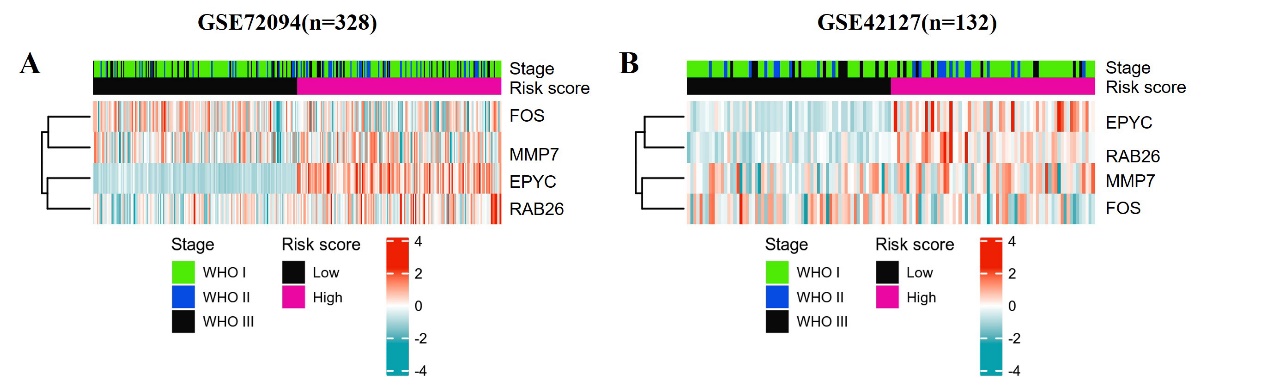


**Supplementary Figure S4** Unsupervised clustering of the 4 candidate genes using the tumour stage, smoking history and risk score as patient annotations in GSE72094 (A) and GSE42127 (B).

**
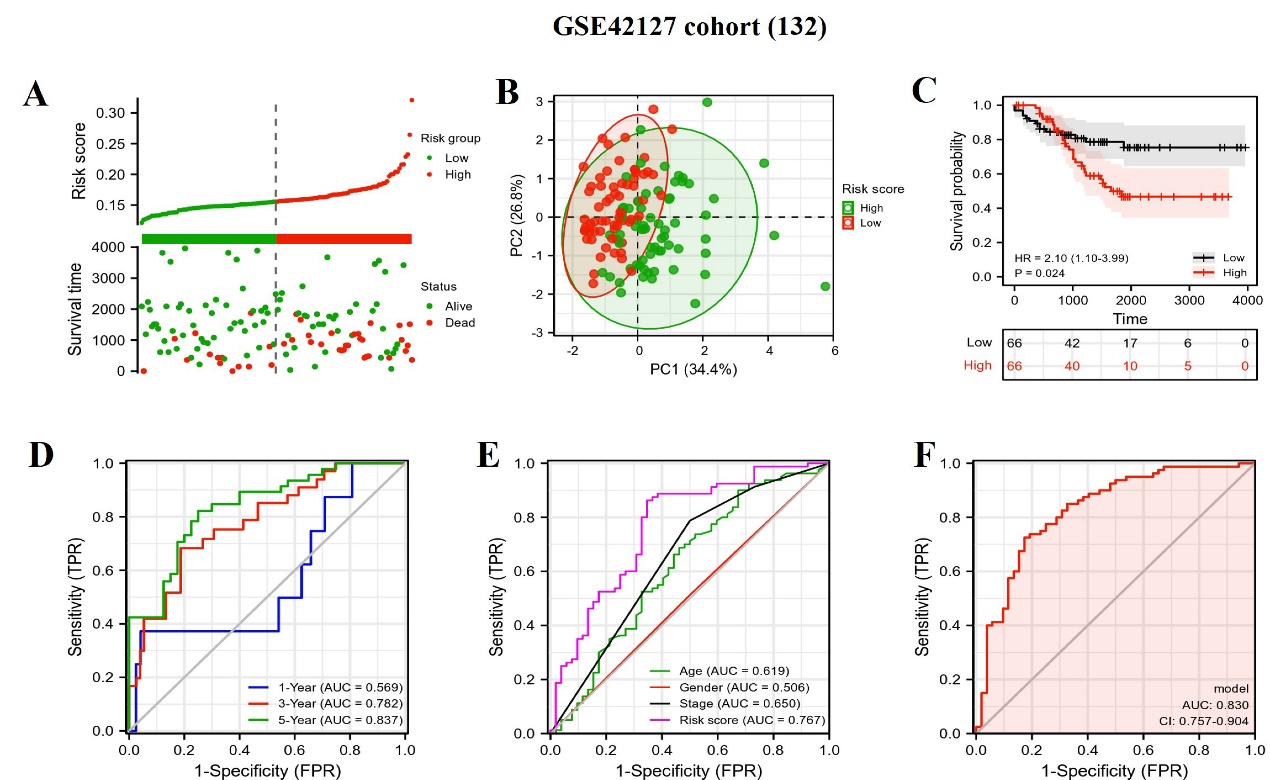
**

**Supplementary Figure S5** Validation of the CTCR model in the GSE42127 dataset (n=132).

(A) Survival status and risk scores in the validation cohort. (B) PCA analysis of grouped samples in the validation cohort. (C) KM curves demonstrating longer OS in the low-risk group. (D) AUC values of time-dependent ROC curves verify the predictive accuracy of the risk score. (E) Comparison of ROC curves among patients with different clinical characteristics, such as age, sex and stage, and risk scores in the validation cohort. (F) ROC curve based on risk scores and other prognostic factors.


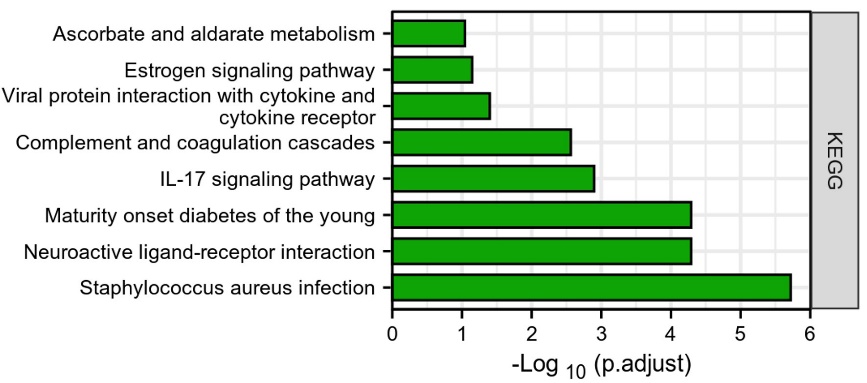


**Supplementary Figure S6** Bar chart derived from KEGG analysis.


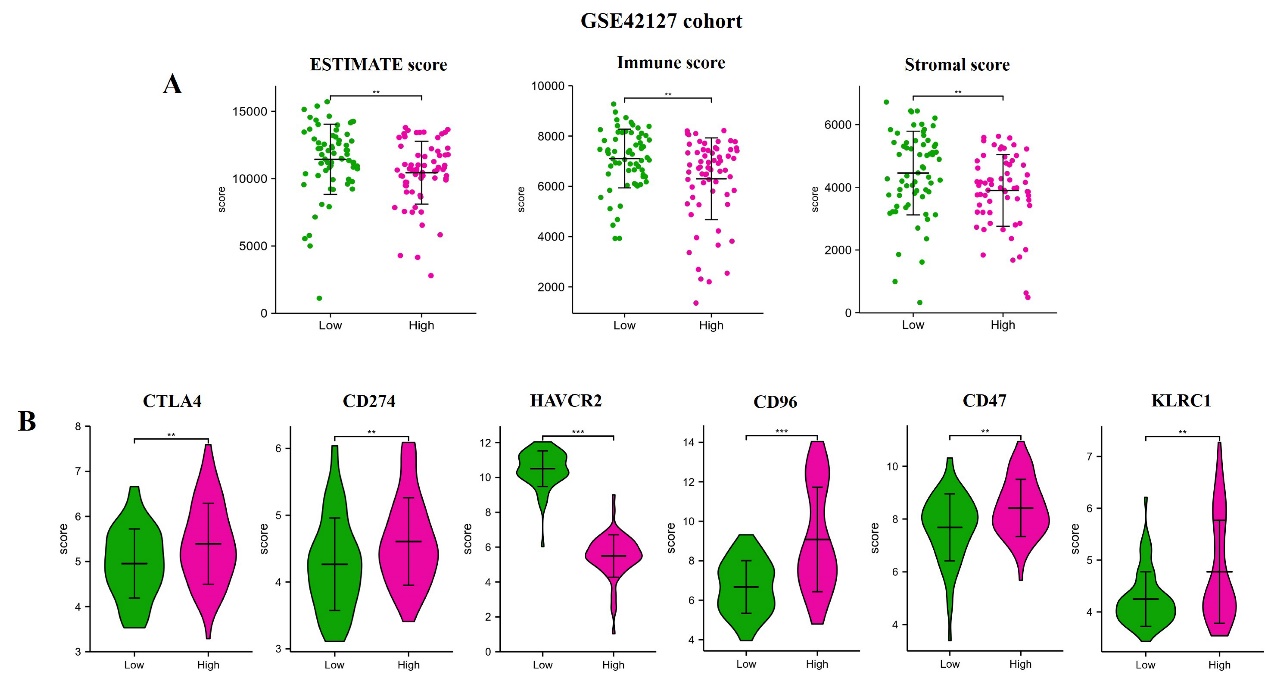


**Supplementary Figure S7** Immune cell infiltration landscape of LUAD in GSE42127 dataset (n=132).

(A) ESTIMATE, immune and stromal scores of high- and low-risk patients. (B) The expression levels of immune checkpoint molecules in the high- and low-risk groups. The asterisks represent the statistical *p* -value (**p <* 0.05; ***p <* 0.01; ****p <* 0.001).
